# Missense Mutations in *FDNC5* Associated with Morphometric Traits and Meat Quality in Hainan Black Goats

**DOI:** 10.3390/ani15040565

**Published:** 2025-02-15

**Authors:** Jing Huang, Mengning Xu, Yuelang Zhang, Jiancheng Han, Hanlin Zhou, Ke Wang

**Affiliations:** 1 Zhanjiang Experimental Station, Chinese Academy of Tropical Agricultural Sciences, Zhanjiang 524013, China; huangjing07182024@163.com (J.H.); xmn07210955@163.com (M.X.); hanjiancheng810@163.com (J.H.); zhouhanlin8@163.com (H.Z.); 2 College of Animal Science and Technology, Guangxi University, Nanning 530004, China; 3 College of Animal Science and Technology, Nanjing Agricultural University, Nanjing 210095, China; 4 Hainan Institute of Zhejiang University, Sanya 572024, China; zhangyuelang@zju.edu.cn; 5 Sanya Research Institute, Chinese Academy of Tropical Agricultural Sciences, Sanya 572024, China

**Keywords:** *FNDC5*, missense mutation, morphometric traits, meat quality, goat

## Abstract

Goats are widely recognized for their adaptability and resource efficiency, making them an excellent choice for sustainable farming. However, the Hainan Black goat (HNBG), a vital breed in southern China’s tropical regions, faces significant challenges that threaten its productivity and economic viability. Specifically, young HNBGs exhibit stunted growth and poor muscle development, indicating the breed may have more genetic defects that cause the poor phenotypes. The *FNDC5* gene, which encodes the protein irisin, plays a key role in promoting mitochondrial biogenesis and oxidative metabolism by activating critical signaling molecules such as PGC-1α, thereby enhancing muscle endurance and metabolic efficiency. This study aimed to investigate the impact of missense mutations in the *FNDC5* gene on growth and meat quality traits in HNBGs. We sequenced a population of HNBGs and identified three SNPs that could lead to amino acid substitutions. Notably, SNP1 (p.119A/V) and SNP2 (p.135R/H) showed strong linkage. Predictions on the structural effects of these mutations indicated that SNP1 (p.119A/V) and SNP3 (p.170W/G) could alter the secondary structure of the FNDC5 protein. Association analyses revealed that SNP1 (p.119A/V) and SNP2 (p.135R/H) were significantly associated with morphometric traits and meat quality. The phenotypic values of SNP1 and SNP2 co-mutants were significantly lower than those of other combined genotypes. Furthermore, gene expression levels of *FNDC5* varied notably across individuals with different SNP1 genotypes. These findings suggest that *FNDC5*-SNP1 (p.119A/V) could serve as a promising genetic marker for selecting HNBGs with improved growth and muscle development, offering a potential pathway for enhancing key economic traits in this breed.

## Simple Summary

Goats are highly adaptable animals that play a key role in sustainable farming, but the Hainan Black goat (HNBG), an important breed in southern China, faces challenges such as slow growth and poor muscle development in young goats. These issues may be linked to genetic defects. This study focused on the *FNDC5* gene, which helps regulate muscle growth and energy metabolism, to understand how genetic variations in this gene affect the growth and meat quality of HNBGs. We identified three genetic changes (mutations) in the *FNDC5* gene, two of which (SNP1 and SNP2) were strongly linked to growth and meat quality traits. Goats with these mutations showed lower growth rates and poorer meat quality compared to others. Additionally, one mutation (SNP1) influenced the activity of the *FNDC5* gene, suggesting it could be used as a genetic marker to select goats with better growth and muscle development. These findings provide valuable insights for breeding programs aimed at improving the productivity and economic value of HNBGs, benefiting farmers and supporting sustainable goat farming in tropical regions.

## 1. Introduction

Goats are an excellent choice for sustainable farming due to their natural adaptability to challenging environments and their ability to thrive with minimal resource inputs [1]. They are highly efficient grazers, capable of utilizing low-quality forage and withstanding extreme weather conditions, making them an ideal livestock option for regions with limited agricultural resources. Goat meat is leaner than many other meats, with lower levels of saturated fat and cholesterol, offering a healthier alternative to beef and lamb. Its high protein content and rich flavor also make it a desirable choice for health-conscious consumers seeking a nutritious and environmentally sustainable source of protein [2,3].

The Hainan Black goat (HNBG), a staple meat breed in tropical southern China, is characterized by its delicate meat and special flavor [4,5], but is currently facing several challenges related to health and productivity [6]. Young goats of this breed often show signs of stunted growth, especially poor muscle development, which are indicative of nutritional deficiencies and suboptimal growth conditions. These health issues contribute to lower meat yields, reduced feed conversion rates, and weakened physical resilience, ultimately affecting the breed’s overall economic value [7,8].

The *FNDC5* gene encodes the fibronectin type III domain-containing 5 protein, and the out-membrane part of this protein, irisin, has garnered significant attention from researchers [9]. Irisin is a myokine released during exercise that promotes the browning of white adipose tissue, enhancing energy expenditure and improving metabolic health [10,11]. In mice, knockout of *FNDC5* not only leads to skeletal muscle damage [12] but also disrupts normal osteocytic osteolysis and osteoclastic bone resorption [13]. FNDC5/irisin deficiency in aged mice exacerbates skeletal muscle wasting, and *FNDC5* expression closely correlates with muscle fiber types in porcine longissimus dorsi muscle [14,15]. Cheng et al. identified *FNDC5* as a production-performance-related and meat-quality-related differentially expressed gene (DEG) through transcriptome sequencing of the longissimus dorsi muscle in different sheep breeds [16]. In our previous transcriptome sequencing study of muscle tissues from Hainan Black goats, *FNDC5* was also identified as a meat-quality-related DEG, and several missense mutations were found in the *FNDC5* gene of goats [17].

Therefore, we hypothesize that, as a production-performance-related and meat-quality-related differentially expressed gene, the missense mutations in *FNDC5* may affect the gene’s expression and influence morphometric traits and meat quality. This study primarily investigates the possible effects of missense mutations in *FNDC5* on growth and meat quality, with the aim of providing insights for meat quality improvement in goats.

## 2. Materials and Methods

### 2.1. Sample Collection

Blood samples were randomly collected from 800 female goats from the Danzhou Hainan Goat Breeding Farm under identical feeding and management conditions [18]. The morphometric traits, including body height (BH, cm), body oblique length (BOL, cm), chest circumference (CC, cm), body weight (BW, kg), and cannon circumference (CAC, cm), were measured and recorded for all 800 goats. From this cohort, 98 two-year-old individuals were randomly selected for slaughter, and various meat quality traits were evaluated, such as carcass weight (CW, kg), longissimus dorsi cross-sectional area (CALM, cm^2^), water loss rate (WLR, %), water-holding capacity (WHC, %), and shear force (SF, N) [19]. Additionally, tissue samples from the heart, liver, brain, skin, cerebellum, uterus, rumen, longissimus dorsi muscle, and gluteofemoral biceps were obtained from 18 adult female goats. Longissimus dorsi muscle samples (*n* = 30) were collected at ages of 0 day, 0.5 year, 1 year, 2 years, and 4 years for expression profiling. At the same time, longissimus dorsi muscle samples (*n* = 32) from 2-year-old females were also used to evaluate the effects of different genotypes of missense mutations on *FNDC5* gene expression levels.

### 2.2. Total RNA and DNA Extraction

Total RNA was extracted using the Trizol method (Solarbio, Beijing, China), and cDNA was synthesized with the PrimeScript™ RT Reagent Kit (Takara, Tokyo, Japan) following the manufacturer’s instructions. Genomic DNA was isolated from ear tissue using the Animal Tissues/Cells Genomic DNA Extraction Kit (Solarbio, Beijing, China), and its concentration was measured with a Nanodrop One spectrophotometer (Thermo Fisher, Waltham, MA, USA). The DNA was diluted to 20 ng/µL and stored at −20 °C.

### 2.3. Primer Design

According to the 18 HNBGs’ RNA-seq results [17] (Supplementary Materials, File S1) and GGVD (Goat Genome Variation Database, http://animal.omics.pro/code/index.php/GoatVar, accessed on 6 May 2024), two pairs of primers were designed to detect five potential missense mutations using the Primer-BLAST tool (http://www.ncbi.nlm.nih.gov/tools/primer-blast/, accessed on 6 May 2024, chr2:14910237 T>G; chr2:14910344 G>A; chr2:14910389 C>T; chr2:14912574 T>C; chr2:14912598 C>T). A pair of primers spanning exons was designed to detect the expression level of *FNDC5* mRNA, with *GAPDH* used as the reference gene (Table S1). Polymerase chain reaction (PCR) and real-time PCR amplification were conducted according to Chen et al. [20], and sequencing was performed following the method described by Wang et al. [21].

### 2.4. Statistical Analysis

Population genetic parameters, Hardy–Weinberg equilibrium (HWE), the polymorphism information content (PIC), and linkage disequilibrium structure were calculated by the Msrcall program (http://www.msrcall.com/Gdicall.aspx, accessed on 17 July 2024) and the GENEPOP (https://genepop.curtin.edu.au/ accessed on 17 July 2024) [22]. The conserved and evolutionary relationships of the *FNDC5* gene across species were compared using the Ensemble gene-tree and orthologous functions. The Chou–Fasman method (https://assets.detaibio.com/tools/chou-fasman-forecast.html, accessed on 2 June 2024) and PredictProtein (Version 1.2.0, https://predictprotein.org/, accessed on 2 June 2024) were used for the prediction of the protein structure. Association tests between the mutations in *FNDC5* and the traits were conducted by utilizing a generalized linear model that was implemented in the SPSS software (Version 18.0, IBM, Armonk, NY, USA), as follows:

Y=μ+G+e

where Y is the phenotypic value, μ is the overall population mean, G is the fixed effect of the genotype, and e is the random error [23]. Gene expression levels were quantified by the 2^−ΔΔCt^ method [21].

## 3. Results

### 3.1. Identification of Missense Mutations in the Goat FNDC5 Gene

**Figure 1:**
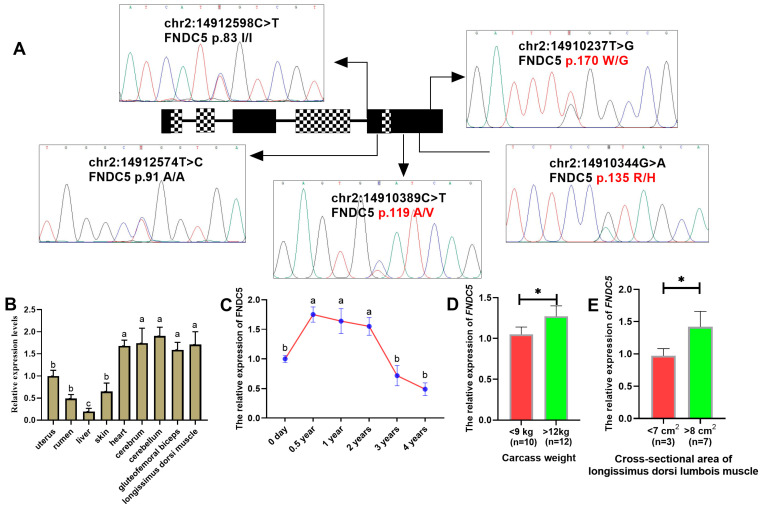
Identification of SNPs in the *FNDC5* gene and expression of the *FNDC5* gene in goats. (**A**) Localization and identification of SNPs in the *FNDC5* gene by Sanger sequencing. The black module indicates the exon region where the two spliceosomes overlap, and the mosaic module represents the region where the two spliceosomes do not overlap. The three missense mutations are marked in red. (**B**) Tissue expression profile of the *FNDC5* gene in adult female goats. Letters (a–c) indicate significant differences (*p* < 0.05) in expression levels among tissues. (**C**) Temporal expression profile of the *FNDC5* gene in longissimus dorsi muscle. Letters (a,b) indicate significant differences (*p* < 0.05) in expression levels across time points. (**D**) Expression of the *FNDC5* gene in individuals with different extreme carcass weights. The asterisk (*) indicates significant differences (*p* < 0.05) between groups. (**E**) Expression of the *FNDC5* gene in individuals with different extreme cross-sectional areas of longissimus dorsi muscle. The asterisk (*) indicates significant differences (*p* < 0.05) between groups.

Among the five mutations detected by Sanger sequencing (Figure 1A), three were identified as missense mutations (chr2:14910389 C>T, *FNDC5* p.119A/V; chr2:14910334 G>A, *FNDC5* p.135R/H; chr2:14910237 T>G, *FNDC5* p.170W/G), while the other two mutations (chr2:14912598 C>T, *FNDC5* p.83I/I; chr2:14912574 T>C, *FNDC5* p.91A/A) were classified as synonymous mutations after alignment with the CDS region of *FNDC5* in Ensembl (https://www.ensembl.org/) and UniProt (https://www.uniprot.org/). The genotypic frequencies of three SNPs are presented in Table 1. All three SNPs exhibited low polymorphism, with SNP1 p.119A/V and SNP3 p.170W/G showing the presence of homozygous mutations. Furthermore, strong interlocking relationships were observed between SNP1 (p.119A/V) and SNP2 (p.135R/H) (Figure S1).

### 3.2. The Impact of Missense Mutations on the Structure of the FNDC5 Protein

**Figure 2:**
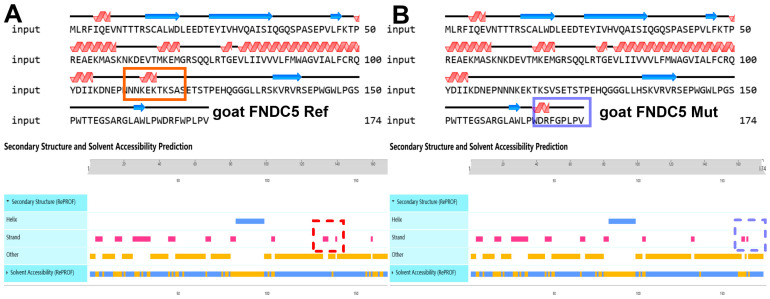
Structural prediction of FNDC5 protein based on reference genome (**A**) and three missense mutations (**B**). The blue arrows represent the strand structure, which forms the beta sheet, and the red waves represent the alpha helix. The blue and red dashed squares show the contrast of secondary structure changes. The orange bars represent other regions where the secondary structure is not predicted. The bar between blue and orange indicates the difference in solvent accessibillity, where blue indicates water affinity and orange indicates fat affinity.

The results of the gene homology analysis indicated that the *FDNC5* gene in goats exhibits the closest genetic relationship with that of other ruminants (sheep, cattle, and other pecora) (Figure S2), which is consistent with the phylogenetic relationships among these species. At the same time, the secondary structure prediction of the FDNC5 protein suggests that the SNP1 (p.119A/V) mutation may result in the loss of the corresponding strand (a linear structure within the β-sheet, Figure 2A), while the SNP3 (p.170W/G) mutation may lead to the formation of a new strand structure at the protein’s C-terminal (Figure 2B).

### 3.3. The mRNA Expression of FNDC5 in HNBGs

The *FNDC5* mRNA expression profiles of adult HNBGs were identified in different tissues. *FNDC5* was expressed in all tissues under evaluation (Figure 1B) and was highly expressed in the brain and muscles, including the heart, cerebrum, cerebellum, gluteofemoral biceps, and longissimus dorsi muscles. The expression of *FNDC5* mRNA in the longissimus dorsi muscle at 0.5, 1, and 2 years of age also showed significant differences compared to 0 days, 3, and 4 years of age (Figure 1C), exhibiting a pattern like individual growth, development, and aging. Furthermore, we found that individuals with extreme phenotypic values for carcass weight (<9 kg vs. >12 kg) and cross-sectional area of longissimus dorsi area (<7 cm^2^ vs. >8 cm^2^) exhibited significant differences in the expression of the *FNDC5* gene.

### 3.4. Association Analysis Between the FNDC5 Missense Mutations and Traits

The genotypic frequencies and population parameters for the three missense mutations are presented in Table 1. No mutant homozygotes were observed for SNP2 (p.135R/H). None of the three mutations conformed to the Hardy–Weinberg equilibrium, and PIC values indicated low levels of polymorphism. It suggests that the genetic diversity of the locus is decreasing due to artificial selection. The association analysis between the traits and the SNPs in the goat *FNDC5* gene revealed that SNP1 (p.119A/V) was significantly correlated with chest circumference, body weight, carcass weight, and the cross-sectional area of the longissimus dorsi lumborum muscle (Table 2). SNP2 (p.135R/H) was significantly associated with chest circumference, body weight, carcass weight, cross-sectional area of the longissimus dorsi lumborum muscle, water loss rate, and water-holding capacity (Table 3). SNP3 (p.170W/G) was significantly associated with carcass weight only (Table S2).

### 3.5. Missense Mutations Haplotype and Combination Genotype Analysis of the FNDC5 Gene

To assess the potential combined effect of the three missense mutations on the phenotype, haplotype and combined genotype analyses were conducted. The results revealed six haplotypes in the tested population. The reference allele haplotype, CGT, was the most prevalent, accounting for 87.2% of the individuals, while the SNP2 single mutation haplotype, CAT, was the least frequent, observed in only 1.1% of the population (Table 4). Six combined genotypes of missense mutations were identified; however, the CCGGGG genotype did not meet the minimum sample size required for association analysis. Among the remaining five combined genotypes, significant differences were observed in phenotypic traits such as body oblique length, chest circumference, body weight, carcass weight, cross-sectional area of the longissimus dorsi muscle, and water loss rate. Notably, individuals with the TTGATT genotype exhibited lower phenotypic values compared to those with other combined genotypes (Table 5).

### 3.6. Missense Mutations Affect the Expression of the FNDC5 Gene

**Figure 3:**
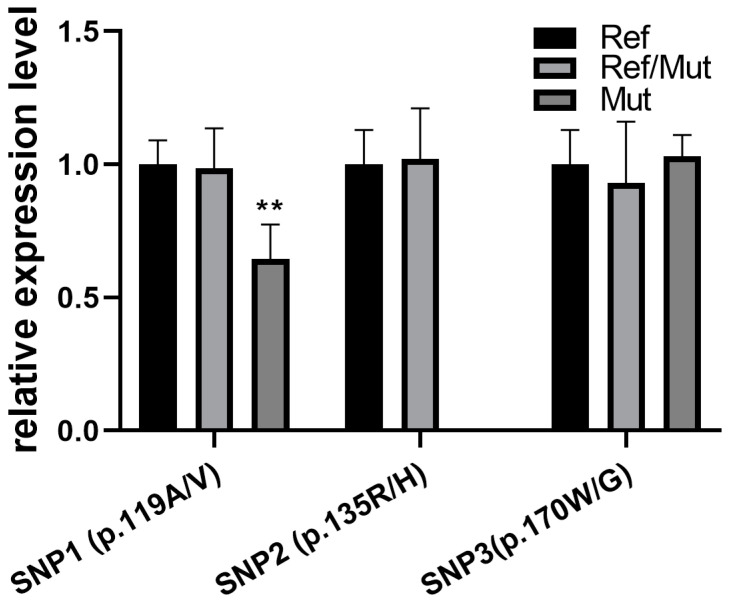
Missense mutations affect the expression of the *FNDC5* gene in goat longissimus dorsi muscle. Comparison with normal genotype; ** indicates *p* < 0.01.

The relationships among different SNP genotypes and mRNA expression levels were detected in female goats’ longissimus dorsi muscle (Figure 3). The different genotypes of SNP1 (p.119A/V) were able to affect *FNDC5* expression (*p* < 0.01), even though SNP2 (p.135R/H) and SNP3 (p.170W/G) showed no significant effects. Particularly, with the mutant homozygote of SNP1 (p.119A/V), *FNDC5* expression showed a 0.65-fold decrease compared with the normal genotype.

## 4. Discussion

*FNDC5* encodes the protein irisin, which promotes mitochondrial biogenesis and oxidative metabolism by activating key signaling molecules such as PGC-1α, enhancing muscle endurance and metabolic efficiency [11,24]. Irisin also activates AMPK, a crucial energy sensor, optimizing energy utilization in muscle cells by increasing fatty acid oxidation and glucose consumption [25,26]. This improves muscle efficiency during prolonged or intense physical activity. Irisin may also influence the interaction between muscle and adipose tissue, promoting the browning of white adipose tissue and increasing fat oxidation, indirectly enhancing muscle energy supply [27]. In our study, *FNDC5* was highly expressed in the brain and muscles, with muscle expression increasing during growth and development but decreasing with aging, consistent with changes in muscle development and energy metabolism. Individuals with extreme phenotypic values showed significant differences in *FNDC5* expression, suggesting a potential dose effect between its expression and muscle development.

The mRNA of the goat *FNDC5* gene has two splice variants, and the three identified missense mutations are located within the overlapping exon regions of these variants. Functional mutations closer to the 5′ end of the coding sequence (CDS) typically have a greater impact. SNP3 (p.170W/G) is located at the end of the CDS, encoding the fourth-to-last amino acid. Different genotypes of SNP3 did not result in differential *FNDC5* mRNA expression, and association analysis showed it was only significantly associated with carcass weight. Although this mutation may alter the secondary structure of the FNDC5 protein, its practical application appears limited. In contrast, SNP1 (p.119A/V) and SNP2 (p.135R/H) showed strong linkage. No mutant homozygotes were detected for SNP2, and different genotypes of SNP2 did not result in differential *FNDC5* mRNA expression or changes in protein structure. Therefore, the phenotypic effects of SNP2 are likely due to its strong linkage with SNP1. Combined genotype analysis revealed that individuals with the TTGATT genotype exhibited significantly lower phenotypic values compared to other genotypes, which included the SNP1 mutant homozygous variant, the SNP2 mutant heterozygous variant, and the normal allelic composition of SNP3. These findings support our hypothesis.

Missense mutations can influence gene expression and protein function through various mechanisms. They can alter the amino acid sequence of proteins, potentially modifying their three-dimensional structure and biological activity, leading to a loss or gain of function and disrupting cellular pathways [28]. Missense mutations can also affect transcription by altering DNA sequences that serve as binding sites for transcription factors, modulating gene expression. They may influence RNA stability and splicing, leading to changes in mRNA levels and translation efficiency [29,30]. Additionally, missense mutations can disrupt protein–protein interaction networks, potentially dysregulating key signaling pathways and gene expression patterns [31]. SNP1 (p.119A/V) is likely to influence the transcriptional process of the *FNDC5* gene, resulting in reduced mRNA expression. In contrast, SNP2 (p.135R/H) and SNP3 (p.170W/G) may exert their regulatory effects through translation or post-translational modifications. Further experimental studies on the functional mechanisms of the mutations are needed to confirm these hypotheses.

According to the above results and analysis, the individual phenotype of the CCGGTT combination genotype is the best. The decreased expression of the FNDC5 gene caused by the SNP1 mutation is not conducive to muscle growth and development. Considering population genetic parameters and PIC, it is expected that differences in phenotypic values caused by mutations can be eliminated by artificial selection.

## 5. Conclusions

This study identified three missense mutations (SNP1, SNP2, and SNP3) in the *FNDC5* gene of Hainan Black goats, with SNP1 (p.119A/V) and SNP2 (p.135R/H) being significantly associated with growth and meat quality traits. SNP1 (p.119A/V) influenced *FNDC5* expression levels and may serve as a genetic marker for selecting goats with improved growth and muscle development. Structural predictions suggest that these mutations, particularly SNP1 and SNP3, could affect the FNDC5 protein’s secondary structure. These findings highlight the potential of *FNDC5*-SNP1 (p.119A/V) for breeding programs aimed at enhancing HNBG productivity.

## Figures and Tables

**Table 1:** Genotypic frequencies and population parameters in *FNDC5*.

Loci	Size	Genotypic Frequencies			HWE *p*-Value	Population Parameters			
	*n*	Ref	Ref/Mut	Mut		Ho	He	Ne	PIC
SNP1 p.119A/V	792	0.866	0.128	0.006	*p* < 0.0.5	0.870	0.130	1.150	0.122
SNP2 p.135R/H	795	0.883	0.117	-	*p* < 0.0.5	0.890	0.110	1.124	0.104
SNP3 p.170W/G	790	0.876	0.118	0.006	*p* < 0.0.5	0.884	0.116	1.132	0.110

Note: HWE, Hardy–Weinberg equilibrium. Ho, observed homozygosity. He, heterozygosity. Ne, effective allele numbers. PIC, polymorphism information content.

**Table 2:** The association analysis between the traits and SNP1 p.119A/V in the goat *FNDC5* gene.

Traits	Genotypes (Mean ± SE)			*p*-Values
	Ref	Ref/Mut	Mut	
body height (cm)	52.24 ± 0.14	52.07 ± 0.19	51.88 ± 0.14	0.317
body oblique length (cm)	54.67 ± 0.21	54.72 ± 0.14	54.92 ± 0.18	0.288
chest circumference (cm)	59.07 ^a^ ± 0.19	58.88 ^a^ ± 0.21	57.79 ^b^ ± 0.12	0.034
body weight (kg)	20.29 ^a^ ± 0.27	19.31 ^b^ ± 0.18	19.57 ^b^ ± 0.07	0.011
cannon circumference (cm)	7.27 ± 0.05	7.19 ± 0.03	7.23 ± 0.04	0.152
carcass weight (kg)	9.71 ^a^ ± 0.19	9.84 ^a^ ± 0.23	9.01 ^b^ ± 0.15	0.027
cross-section area of longissimus dorsi *lumbois* muscle (cm^2^)	7.84 ^a^ ± 0.17	7.75 ^a^ ± 0.06	7.24 ^b^ ± 0.12	0.043
water loss rate (%)	4.85 ± 0.10	4.88 ± 0.24	4.77 ± 0.31	0.302
water-holding capacity (%)	4.82 ± 0.24	4.78 ± 0.13	4.84 ± 0.16	0.424
shear stress (N)	48.09 ± 0.37	48.38 ± 0.22	47.59 ± 0.41	0.087

Note: letters (a,b) indicate significant differences (*p* < 0.05) between different genotypes and traits.

**Table 3:** The association analysis between the traits and SNP2 p.135R/H in the goat *FNDC5* gene.

Traits	Genotypes (Mean ± SE)			*p*-Values
	Ref	Ref/Mut	-	
body height (cm)	51.64 ± 0.30	51.97 ± 0.14		0.425
body oblique length (cm)	54.73 ± 0.25	54.34 ± 0.10		0.174
chest circumference (cm)	57.94 ± 0.13	57.23 ± 0.28		0.027
body weight (kg)	19.99 ± 0.24	19.27 ± 0.09		0.043
cannon circumference (cm)	7.18 ± 0.04	7.21 ± 0.05		0.684
carcass weight (kg)	9.75 ± 0.24	9.31 ± 0.26		0.058
cross-section area of longissimus dorsi *lumbois* muscle (cm^2^)	7.87 ± 0.22	7.11 ± 0.29		0.007
water loss rate (%)	4.95 ± 0.21	4.31 ± 0.17		0.041
water-holding capacity (%)	4.83 ± 0.14	4.69 ± 0.19		0.026
shear stress (N)	49.10 ± 0.26	48.98 ± 0.37		0.073

**Table 4:** The frequency analysis of the goat *FNDC5* gene haplotype.

SNP Haplotype	SNP1 p.119A/V C>T	SNP2p.135R/H G>A	SNP3p.170W/G T>G	Frequency
HI	C	G	T	0.872
H2	C	G	G	0.046
H3	C	A	T	0.011
H4	T	A	T	0.032
H5	T	A	G	0.020
H6	T	G	T	0.019

**Table 5:** The association analysis between the traits and *FNDC5* SNPs’ combined genotype.

Combined Genotype	BH (cm)	BOL (cm)	CC (cm)	BW (kg)	CAC (cm)	CW (kg)	CALM (cm^2^)	WLR (%)	WHC (%)	SS (N)
CCGGTT	51.79 ± 0.27	54.83 ^a^ ± 0.32	58.78 ^a^ ± 0.37	20.09 ^a^ ± 0.27	7.21 ± 0.08	9.81 ^a^ ± 0.20	7.80 ^a^ ± 0.29	4.73 ^a^ ± 0.18	4.68 ± 0.24	48.89 ± 0.23
CCGGTG	51.82 ± 0.19	54.71 ^a^ ± 0.27	58.69 ^ab^ ± 0.25	19.88 ^a^ ± 0.31	7.20 ± 0.07	9.68 ^a^ ± 0.33	7.73 ^a^ ± 0.29	4.69 ^a^ ± 0.21	4.72 ± 0.18	49.01 ± 0.15
CCGATT	51.77 ± 0.34	54.68 ^ab^ ± 0.38	58.47 ^b^ ± 0.21	19.75 ^a^ ± 0.28	7.23 ± 0.09	9.72 ^a^ ± 0.14	7.88 ^a^ ± 0.29	4.80 ^a^ ± 0.12	4.62 ± 0.31	48.79 ± 0.38
CTGATT	51.62 ± 0.22	54.75 ^a^ ± 0.40	58.23 ^bc^ ± 0.41	19.64 ^ab^ ± 0.16	7.22 ± 0.09	9.60 ^ab^ ± 0.19	7.28 ^ab^ ± 0.29	4.73 ^a^ ± 0.25	4.42 ± 0.27	48.88 ± 0.27
TTGATT	51.80 ± 0.43	54.70 ^a^ ± 0.38	57.62 ^c^ ± 0.53	19.48 ^b^ ± 0.38	7.19 ± 0.21	8.95 ^b^ ± 0.49	7.01 ^b^ ± 0.41	4.27 ^b^ ± 0.42	4.66 ± 0.36	48.75 ± 0.51

Note: letters (a–c) indicate significant differences (*p* < 0.05) between different genotypes and traits.

## Data Availability

The original contributions presented in this study are included in the article/Supplementary Materials. Further inquiries can be directed to the corresponding author.

## Supplementary Materials

The following supporting information can be downloaded at: https://www.mdpi.com/article/10.3390/ani15040565/s1, Figure S1: Heat map of linkage disequilibrium analysis of missense mutations in goat *FNDC5* gene; Figure S2: Ensembl gene-trees of the *FNDC5* gene; Table S1: Primer information; Table S2: The association analysis between the traits and SNP3 p.170W/G in the goat *FNDC5* gene; File S1: List of SNPs obtained by transcriptome sequencing annotation of HNBG.
