# Supplementary material for: Missense Mutations in FDNC5 Associated with Morphometric Traits and Meat Quality in Hainan Black Goats
Source: Animals (Basel). 2025 Feb 15;15(4):565. doi: 10.3390/ani15040565 (PMC11851529; doi:10.3390/ani15040565)
Supplement: Supplementary file 1 [file animals-15-00565-s001.zip › Table S1 and S2.pdf]

Supplementary Tables

Table S1. Primer information

**Gene name**		**5'-3'**	**Length**	**Note**
FNDC5	F	AAGTCCAGTTACTGCCCCCT	895	Chr2:14912119-14913013
	R	CACGGATTGGGAGGCTTCAT		
	F	CTGCATCAGAGCGGGTAAGA	462	Chr2:14910137-14910599
	R	CTGCACTCTGGTTAAACCCCT		
FNDC5	F	TGGCCTCGAAGAACAAAGATGAG	275	mRNA-qPCR
	R	CAGAGCAAGCACTGAAAGGTT		
GADPH	F	TGAAGGTCGGTGTGAACGGATTTGG	277	mRNA-qPCR
	R	ACGACATACTCAGCACCAGCATCAC		

Table S2. The association analysis between the traits and SNP3 p.170W/G in the goat *FNDC5* gene.

Traits	Genotypes (Mean ± SE)			*p* values
	Ref	Ref/Mut	Mut	
body height (BH,cm)	51.74±0.24	51.97±0.29	51.65±0.10	0.355
body oblique length (BOL, cm)	54.37±0.25	54.76±0.10	55.12±0.08	0.210
chest circumference(CC, cm)	57.94±0.12	56.88±0.17	58.09±0.22	0.082
body weight (BW, kg)	18.99±0.24	19.27±0.09	20.31±0.09	0.061
cannon circumference(CAC, cm)	7.17±0.05	7.19±0.01	7.33±0.04	0.104
**Carcass weight (CW, kg)**	**9.61^b±0.41**	**9.49^b±0.36**	**9.85^a±0.22**	**0.044**
cross-section area of *longissimus dorsi lumbois* muscle(CALM, cm^2)	7.54±0.19	7.78±0.26	7.92±0.35	0.107
water loss rate (WLR, %)	4.79±0.05	4.82±0.14	4.91±0.22	0.606
water holding capacity (WHC, %)	4.75±0.27	4.89±0.15	4.84±0.09	0.519
shear stress (SS, N)	48.51±0.29	47.97±0.27	47.33±0.29	0.277
