# Supplementary figures and images for: Missense Mutations in FDNC5 Associated with Morphometric Traits and Meat Quality in Hainan Black Goats
Source: Animals (Basel). 2025 Feb 15;15(4):565. doi: 10.3390/ani15040565 (PMC11851529; doi:10.3390/ani15040565)

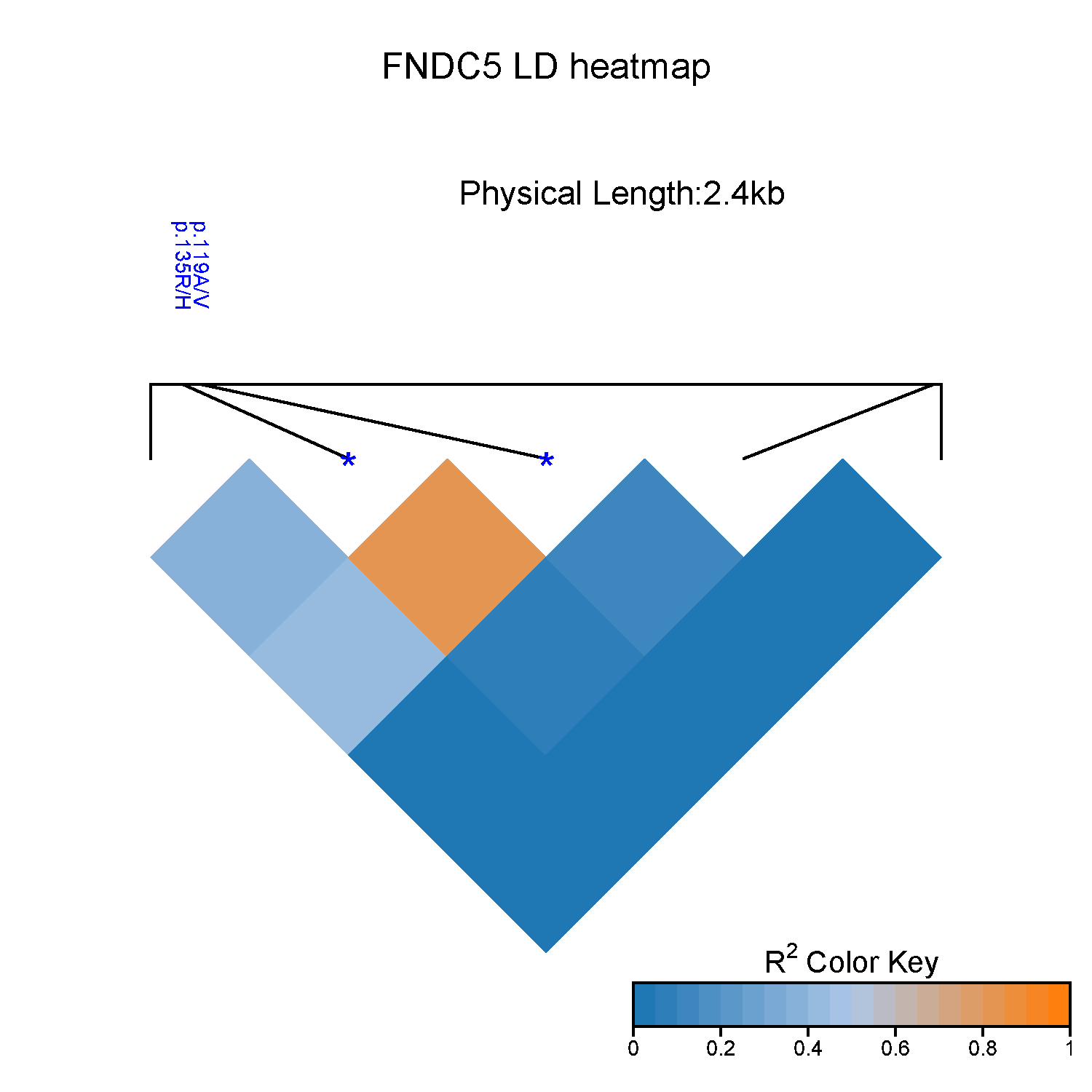

Supplement: Supplementary file 1 [file animals-15-00565-s001.zip › Figer S1.png]

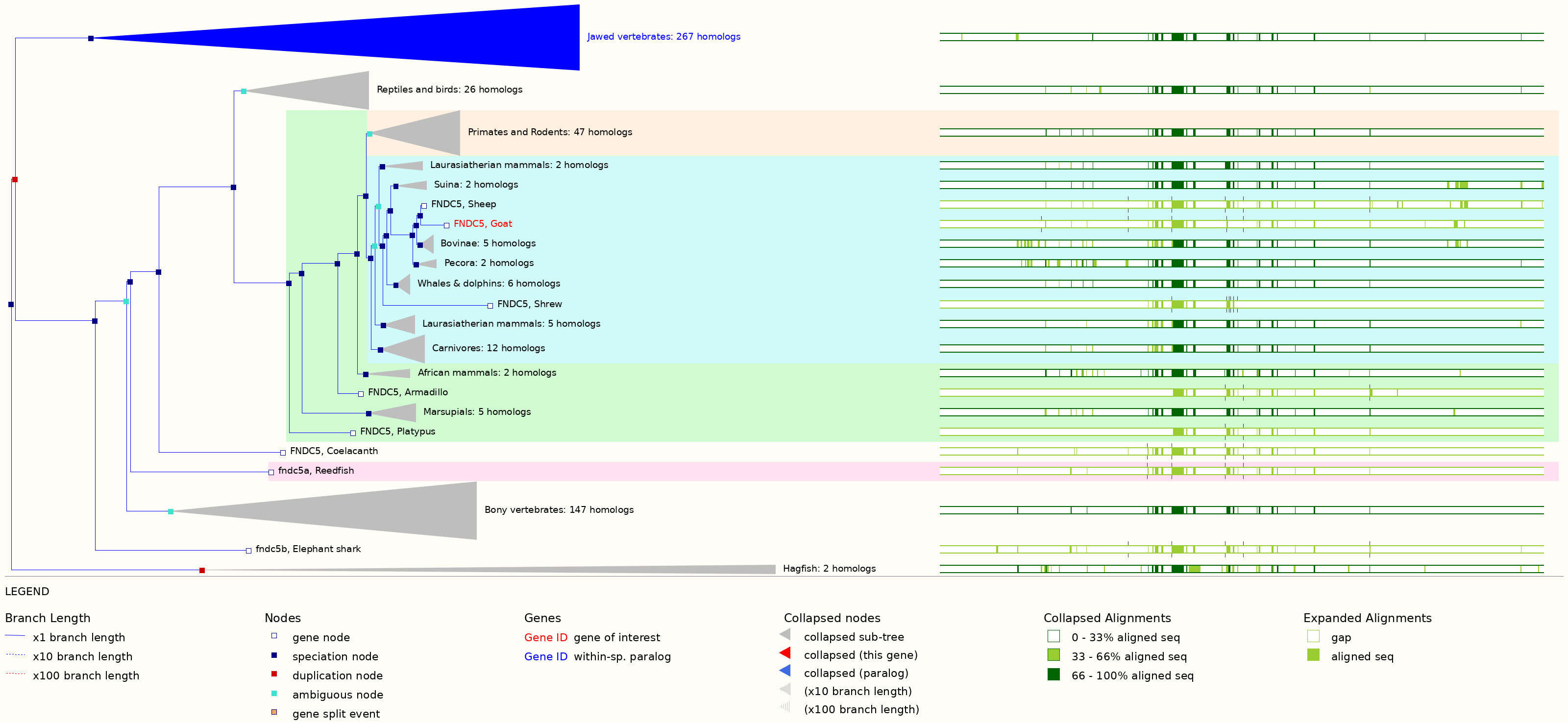

Supplement: Supplementary file 1 [file animals-15-00565-s001.zip › Figure S2 Goat_FNDC5.png]
